# Comparative analysis of solar still with and without use of coating of waste toner powder on absorber plate and hyperbolic fins

**DOI:** 10.1038/s41598-025-23021-y

**Published:** 2025-12-26

**Authors:** Pradeep Boka, Choon Kit Chan, Nithesh Naik, Subhav Singh, Deekshen Varshney, Md Irfanul Haque Siddiqui

**Affiliations:** 1 Department of Mechanical Engineering, Government Engineering College, Palanpur, 385001 Gujarat India; 2 Faculty of Engineering and Quantity Surveying, INTI International University, Nilai, Negeri Sembilan 71800, Malaysia; 3 Department of Mechanical and Industrial Engineering, Manipal Institute of Technology, Manipal Academy of Higher Education, Manipal, 576104 Karnataka India; 4 Centre of Research Impact and Outcome, Chitkara University, Rajpura, 140417 Punjab India; 5 Centre for Promotion of Research, Graphic Era (Deemed to be University), Uttarakhand, Dehradun, India; 6 Division of research and development, Lovely Professional University, Phagwara, Punjab India; 7 Department of Mechanical Engineering, College of Engineering, King Saud University, Riyadh, 12372 Saudi Arabia

**Keywords:** CSS, Distillate yield, MSS, Payback time, Solar still, Energy efficiency, Energy science and technology, Engineering, Environmental sciences

## Abstract

Solar stills are considered to be a simple apparatus used to convert saline water into potable water by the application of solar energy. Because of its low distillate yield, it is not used for industrial or domestic applications of potable water. This study introduces a modified solar still (MSS) that integrates a toner waste powder (TWP) nanocoating with composite hyperbolic fins, tested under the climatic conditions of Palanpur, Gujarat, India. Both fully and partially submerged fins were examined, and a partially submerged design showed superior thermal performance. Compared to the conventional solar still (CSS), the MSS achieved ~ 4 °C higher basin water temperature, yielding 45.84% more freshwater (0.999 L vs. 0.685 L per 0.25 m^2^), energy and exergy efficiencies increased by 45.6% and 72%, respectively. Despite 36% higher manufacturing costs, unit water cost decreased (0.0148 vs. 0.0158 USD/L), while payback time was shortened (3.76 vs. 4.02 months). These findings demonstrate that hyperbolic fins integrated with waste-derived nanocoating are a cost-effective and sustainable alternative to conventional nanomaterials for solar desalination.

## Introduction

Water is one of the most important resources of life on Earth. People used it for drinking, hygiene, farming, industry, and the preservation of healthy ecosystems. Although water covers over 71% of the planet’s surface, freshwater accounts for only approximately 2.5% of that. However, much of it is buried in glaciers or deep below them, making it difficult to extract and use directly^1^. The demand for freshwater exceeds the available supply, or when the water quality is unsafe for consumption, it leads to water scarcity. They are divided into two types: physical scarcity, in which natural resources are scarce, and economic scarcity, in which access is restricted by inadequate infrastructure or a lack of resources^2^. Population growth, climate change^3^, pollution, water abuse, and deforestation are key drivers. The implications are serious, including food shortages, health issues^4^, economic losses, environmental harm, and social and political conflicts over scarce water resources.

A solar still is a simple but important device that uses solar energy to make saline or brackish water drinkable. It functions like a natural cycle of evaporation and condensation. Water in a basin heats up from the sun, evaporates, and then condenses on a cooler surface, where it is collected as pure distilled water. Although this method is energy efficient and cost-effective, distillate production is a primary issue^5^. Owing to their lower output, solar still devices currently account for less than 0.65% of overall solar desalination plant capacity^6^.

Improving the performance of solar stills is a critical area of research, with a strong emphasis on increasing the distillate yield from improved heat absorption and retention in the absorber plate. The absorber plate temperature is critical for distillate productivity because it directly affects the water temperature, glass temperature, evaporation rate of water, heat transfer, and overall efficiency of solar stills^7^. An interesting option is the modification of the basin design and nano-coating of the absorber plate, which significantly enhances the heat transfer, heat conductivity, and sun absorption properties.

Recent developments, such as the use of nanoparticles and combined systems, have improved the performance of solar stills by making them better at absorbing and transferring heat. These improvements make solar stills a better and more environmentally friendly way to deal with water shortages, especially in places that are dry and short of water. As the demand for clean water increases, solar stills have become a viable and sustainable solution that integrates renewable energy with water purification.

Numerous studies have examined the use of nanoparticles (NPs) in either absorber plates or basin water to improve production. Kumar et al.^8^ reported that the addition of 50–100 nm Al_2_O_2_ nanoparticles to black paint caused 38.09% more productivity and 12.18% more thermal efficiency. Likewise, Gupta et al.^9^, with the use of CuO particles, demonstrated 22.4% greater distillate yield and 30% greater distillate yield at 5 and 10 cm water depths, respectively. CuO NP-coated sprinkler systems and water basins showed a 37.9% improvement in distillate yield^10^.

The use of various nanoparticle materials has led to notable thermal improvement. TiO_2_-coated absorber plates raised water temperature by 1.5 °C and improved distillate output by 6.1%^11^. Meanwhile, ZnO nanoparticles increase thermal efficiency from 21% to 30% in unmodified stills^12^. Reduced graphene oxide (RGO) combined with activated carbon pellets enhances distillate yield by 58.15% and energy efficiency by 64.44%^13^.

Metallic coatings have several advantages. Han et al.^14^ discovered that a 15 wt% Si coating led to temperatures that were 65.81% higher than those of bare aluminum plates. In the same way, 0.5% Nano-SiO_2_ coated absorber plate increased distillate output by 8.78% and 7.83% for 15 mm and 25 mm water depths respectively, compared with solar still without coating^15^. Metallic Zn combined with black paint showed a 17.57% improvement in indoor tests^16^. SnO₂ coatings reached a surface temperature of 101.61 °C, which is a 53.67% increase compared to uncoates surfaces^17^. Enhancement of conventional solar stills (CSS) by coating the absorber surface with cobalt ferrite (CoFe_2_O_4_) nanoparticles to improve the energy conversion and showed a 66.7% increase in freshwater yield and a 14.7% increase in water temperature compared to CSS^18^.

Some studies examined combinations of improvements. Abdullah et al.^19^ found that using reflectors, CuO nanoparticles, and PCM together increased output by 108% compared with the standard method. Changes in multiple features, including ISR, PCM, Al_2_O_2_-PCM, and HCF, led to an improvement of up to 51.8%^20^.

Other new methods include oxygen plasma treatment and graphene enhancement^21^, PARC combinations (PCM/Ag/Ricinus Communis)^22^, and silver nanoparticles with paraffin wax^23^. All of these factors led to significant increases in output and efficiency. Nonmetal options, like pistachio shell powder^24^, showed a 46.26% improvement in energy storage materials. Discarded soda cans coated with carbon soot form exhaust as PCM containers, improve solar still output by over 100%^25^ (Table 1).

**Table 1:** Literature study with different nanomaterials.

Authors	Nano material	Configuration/method	Key findings
Kumar et al.^8^	A_2_O_3_	50–100 nm NPs with black paint, single slope still	At 1 cm water depth: 3.48 L, 38.65% efficiency; productivity ↑ 38.09%
Gupta et al^9^	CuO	0.12% CuO in water, depths 5 cm & 10 cm.	Productivity ↑ 22.4% & 30%.
Gupta et al.^10^	CuO	Basin CuO + sprinkler	Output ↑ 37.9%, effectiveness 54.54%.
Kabeel et al.^11^	TiO_2_	Coated absorber in pyramid still	Water temp. ↑ 1.5 °C, output ↑ 6.1%
Panchal et al.^12^	ZnO	ZnO in water drip still	Output 3.04 L/m^2^ vs. 1.887 L/m^2^; efficiency 52.5%
Thakur et al.^13^	RGO	RGO + activated carbon pellets	Output ↑ 58.15%, efficiency ↑ 64.44%
Han et al.^14^	Si	Si in paint (10–15 wt%)	Indoor: +65.81% temp; Outdoor: +6.38% temp
Manoj Kumar et al.^15^	SiO_2_	Nano-SiO₂ coated plate, depths 15 & 25 mm	Output ↑ 8.78% & 7.83%
Ahmad et al.^16^	Zn	Metallic Zn in black paint (10 wt%)	Indoor: 103.53 °C (+ 17.57%); Outdoor: 87.53 °C (+ 9.41%)
Rasachak et al.^17^	SnO_2_	SnO₂ in black paint (0.5–20 wt%)	At 15 wt%: 101.61 °C (+ 53.67% over bare plate).
Pradhan et al.^18^	CoFe_2_O_4_	Coated absorber plate with CoFe₂O₄	Output ↑ 66.7%, efficiency ↑ 38.1%
Abdullah et al.^19^	CuO	CuO paint + PCM + reflectors	Output ↑ 14% (CuO alone), 108% (combined).
Tuly et al.^20^	A_2_O_3_	Al₂O₃-PCM with ISR & HCF	Output ↑ 21.5% over PCM alone.
Baticados et al.^21^	Graphene	Graphene-enhanced plate + plasma treatment	Output ↑ 48.9%
Ellappan et al.^22^	PARC	PCM/Ag/Ricinus Communis (PARC)	Output ↑ 124.2%, efficiency ↑ 52%
Sathyamurthy et al.^23^	Ag	Ag NPs (1–2%) in paraffin wax	Output 7.98 kg/m^2^ vs. 3.61 kg/m^2^
Noman et al.^24^	Pistachio shell powder	Pistachio shell powder	Output ↑ 46.26%
Sathyamurthy et al.^25^	PCM	Carbon soot on PCM cans	Output ↑ 102.3%, thermal ↑ 75.7%.

Panomwan et al.^26^ found that adding plate-type fins to a standard solar still basin improved distillate production by 15.5%. The simulation results also showed a 46% improvement in thermal efficiency owing to increased solar absorption.

El-Sebaii et al.^27^ researched the best fin designs for finned-basin linear solar stills. They found that while increasing fin height increased output, increasing either fin thickness or number decreased output, resulting in an increase of 13.7% in daily output of water using the optimum parameters.

Kabeel et al.^28^ incorporated hollow copper circular fins and a PCM tank located beneath the absorber plate into a pyramid solar still. The distillate output improved by 43%, and the PCM improved by 101.5%. Sathyamurthy et al.^29^ also added fins to a tubular solar still, which improved distillate output by 46.85%.

Kaviti et al.^30^ assessed truncated conic aluminum fins with various water levels and concluded that the maximum output was at the 1 cm level. In a similar study, Kaviti et al.^31^ used parabolic fins and produced an extra 70 mL of distillate compared with the conventional still. Kateshia et al.^32^ showed that the PCM and pin fins produced 30% more than the scenario with only PCM.

Modi et al.^33^ studied hollow aluminum and wick fins using a jute cloth. Wick fins were particularly effective/cheap when partially submerged, with productivity being 8.61% higher than using black cotton fabric wicks. Javad et al.^34^ used 144 hollow copper fins with glass wool insulation in a pyramid solar still, which resulted in 62.5% greater productivity.

Tuly et al.^35^ included various combinations of techniques: hollow fins, PCM, nano-PCM, and reflectors with double-slope stills. At this instantiation, the nano-PCM with reflectors and fins produced the highest overall performance of 92%, better output, and an efficiency of 21.56%.

Ahmed et al.^36^ used cylindrical fins with PCM at different heights, as well as different water levels, inside pyramid stills. Their study provided a fin height of 40 mm, which achieved an efficiencies of 43.4% as well as 89.9% using PCM. Kumar et al.^37^ referenced research on nanoparticle-enhanced heat-storage devices using fins inside a double-slope passive solar still. They showed a production increase of 86.39% with the fins, of which 74.29% of it was with nanoparticles, and well as thermal efficiency and exergy efficiency increased by 41.18% and 59.54%, respectively.

Bady et al.^38^ performed experiments by integrating phosphate-filled copper fins on conical solar distillers and showed 69.8% and 84.6% improvements in distillate output and thermal efficiency, respectively. Ghriss et al.^39^ used square fins for three different fin quantities in double slope solar still and obtained 54.50% higher freshwater with 12 number of fins.

The reviewed studies show that distillate yield in solar stills can be improved by accelerating either evaporation or condensation, most often by enhancing solar absorption through nanoparticle coatings or by increasing heat transfer via fin modifications. However, these strategies are usually applied independently, and very few studies have attempted to integrate surface coatings with advanced fin geometries into a single optimized system. Moreover, the reliance on conventional nanomaterials, such as Al_2_O_2_, CuO, and TiO_2_, although effective, raises concerns about cost, scalability, and environmental impact. A promising but under-explored alternative is the use of waste-derived nanomaterials. Toner waste powder (TWP), a carbon-rich by-product of the printing industry, contains nanoscale pigments with excellent light absorption and thermal conductivity. Despite its abundance and environmental challenges associated with its disposal, TWP has rarely been considered for solar desalination. Similarly, while various fin geometries—straight, parabolic, and hollow—have been investigated, nonlinear designs such as hyperbolic fins, which offer increased surface area and improved thermal distribution, remain largely unexamined.

To address these gaps, this study introduces a Modified Solar Still (MSS) that combines hyperbolic fins with absorber plates coated with TWP nanoparticles. This dual modification provides a synergistic effect: the hyperbolic fins expand the effective heat transfer area, while the TWP coating enhances the solar absorptivity, thermal conductivity, and water heating efficiency. Importantly, this approach also transforms a problematic industrial waste stream into a functional, sustainable resource. Experimental validation demonstrates that this system delivers a 45.84% increase in daily freshwater yield compared to a conventional solar still, underscoring its potential as a low-cost, scalable, and environmentally responsible solution to the global freshwater crisis.

## Materials and methods

### Materials

Toner waste powder (TWP) is a byproduct of printer and photocopier operation. It mainly contains small carbon-based particles, magnetic materials, such as ferrite or magnetite, and polymer binders. TWP has a particle size that ranges from nano-to micro-scale, usually less than 100 nm when processed. This size gives it properties typical of nanomaterials, including large surface area, high reactivity, and strong optical absorption.

TWP’s high carbon content and dark black color of TWP help it absorb a wide range of solar radiation, including visible and near-infrared wavelengths. This makes it an excellent option for solar thermal applications, especially as a coating for absorber plates in solar stills and collectors. The use of TWP nanoparticles as a coating material for the absorber plate of solar stills represents a novel approach for enhancing distillate output.

### Characterisation of TWP NPs

**Fig. 1:**
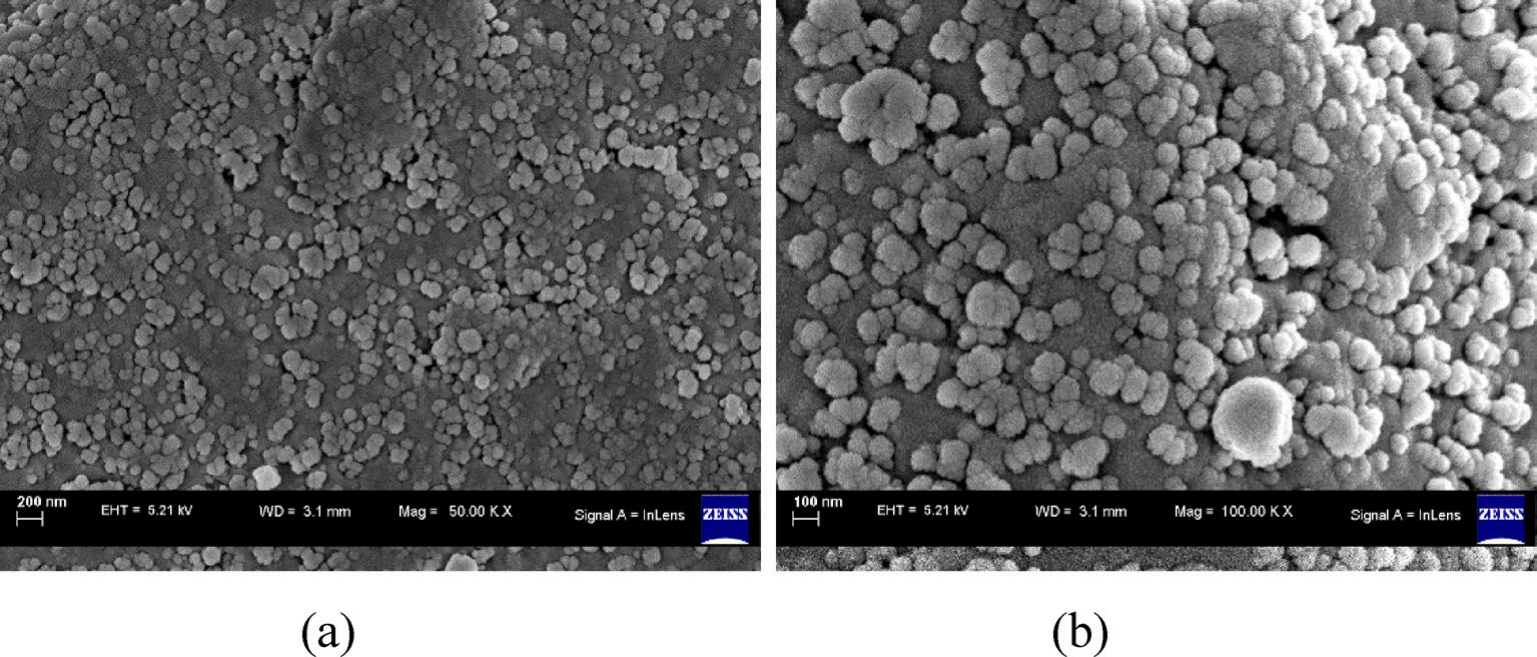
FESEM image analysis of TWP^40^.

**Fig. 2:**
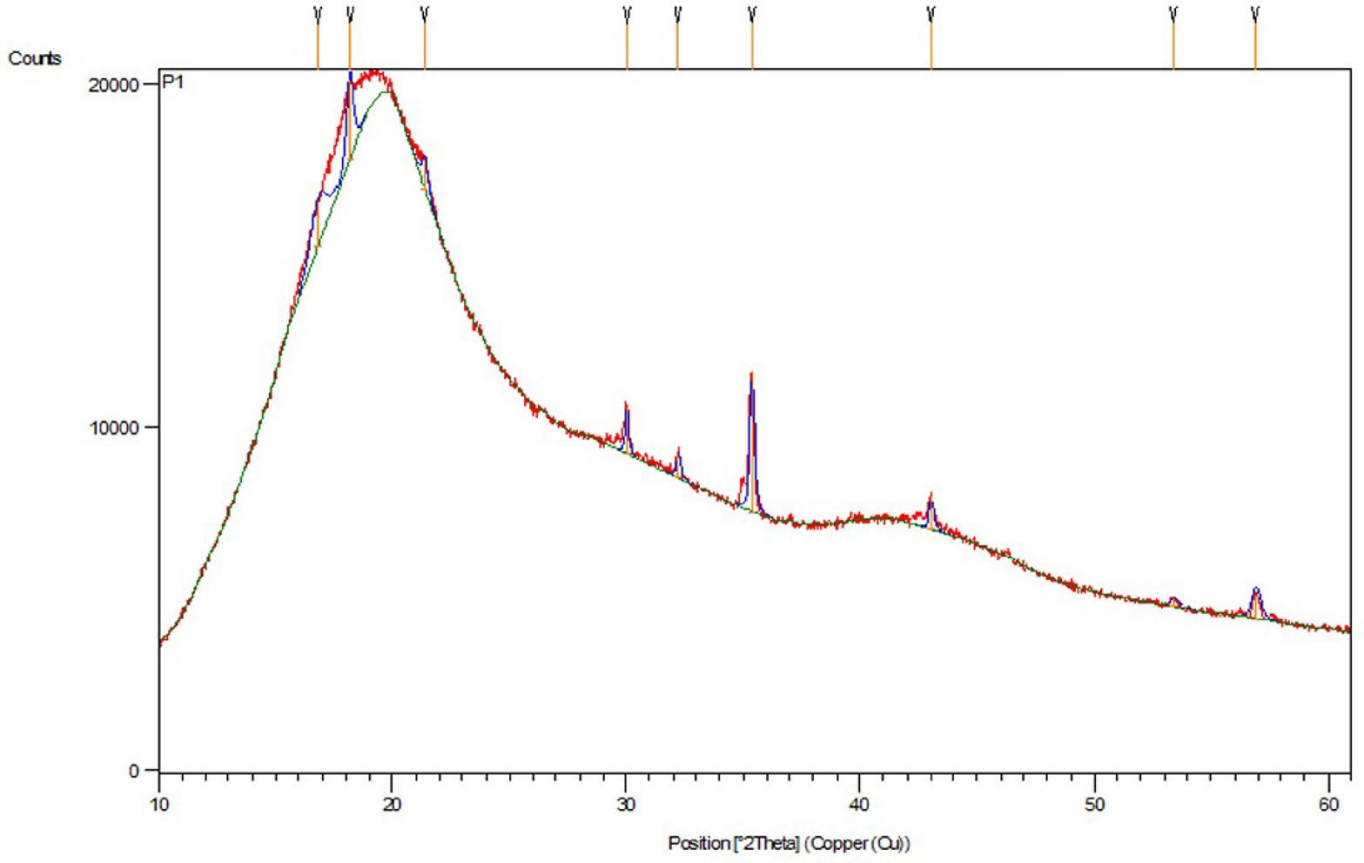
X-ray diffraction pattern of TWP^40^.

Figures 1 and 2 show Field Emission Scanning Electron Microscopy (FESEM) image analysis and X-ray diffraction to characterize the particle morphology and distribution. FE-SEM was performed using a Carl Zeiss Model Supra 55 (Germany), while XRD was performed using a Bruker D8 Focus with a copper anode at 40 kV and 35 mA. The particles had a mean size of 100 nm and ranged from 58 nm to 159 nm. The isotropic microstructural arrangement resulted from the random orientation of the particles, helping to reduce directional reflection losses and increase solar absorptivity^40^.

### Hyperbolic fin design

Performance enhancement of solar stills can be achieved through absorber plate modifications that improve the heat transfer and evaporation rates. Fin integration is a proven approach because it increases the effective heat-exchange surface. Previous studies have predominantly investigated circular and square fin geometries; however, hyperbolic contours have not been reported in the literature.

In this study, a hyperbolic fin profile was conceived and modelled using *SolidWorks 2023*. Surface area calculations were performed and benchmarked against circular and square fins with identical overall dimensions. The hyperbolic configuration yielded the highest surface area, indicating the potential for an improved absorber plate heat flux.

**Fig. 3:**
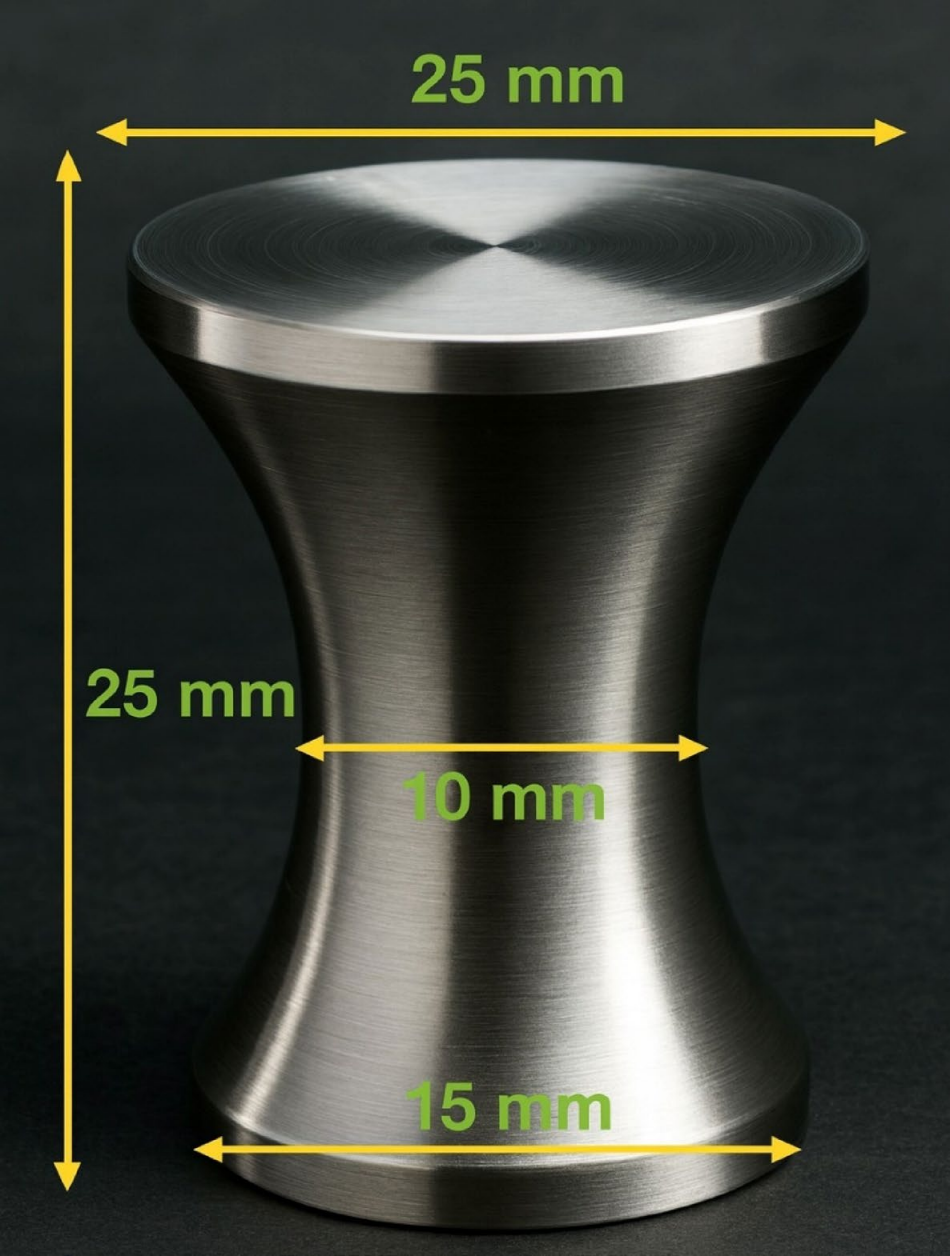
Design of Hyperbolic fin.

**Fig. 4:**
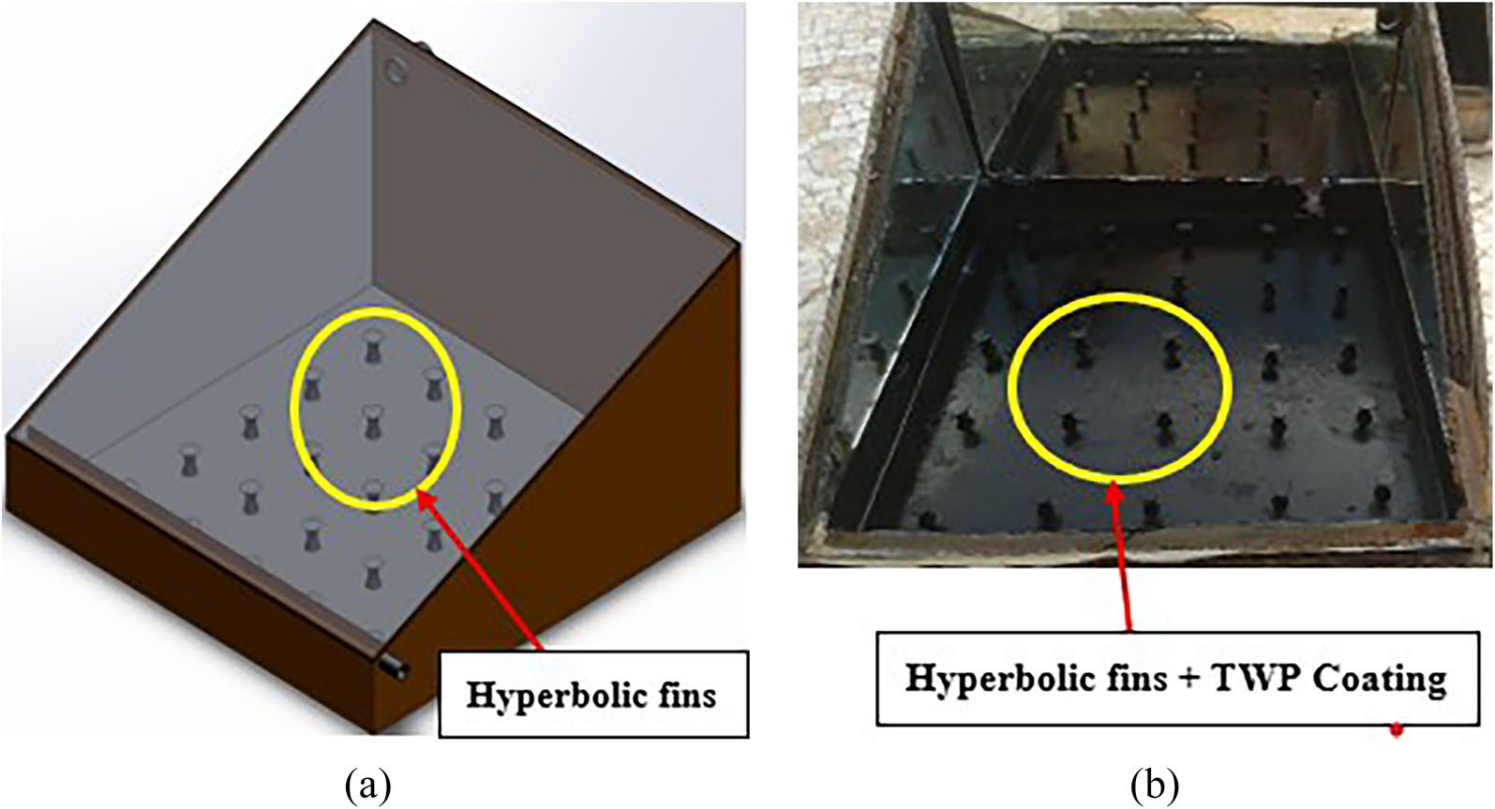
Solar still absorber plate with hyperbolic fins and TWP Coating (**a** CAD model and **b** Actual experimental model).

The fins were fabricated using conventional lathe machining from unused waste iron bars. Each fin measured 25 mm in height, with a base diameter of 15 mm, top diameter of 20 mm, and minimum diameter of 10 mm located in the lower middle section. The detailed geometry is presented in Fig. 3. The fins (25 numbers) were uniformly mounted on the absorber plate in five rows and five columns (5 × 5 matrix) to increase the effective heat-transfer area. The fin-plate assembly is shown in Fig. 4.

### Preparation of absorber plate with fins and coating with TWP NPs

Before coating the TWP NPs on the absorber plate, the TWP NPs were mixed gradually with black paint at a ratio of 50:5 W/W under continuous stirring. At this proportion, solar stills yield higher distillate production^41^. The mixture was stirred for 20 min with a magnetic stirrer to initiate dispersion. The dispersion quality was checked using optical microscopy (100×) to confirm the absence of visible agglomerates.

Turpentine (20.00 g) was added dropwise to adjust the viscosity under continuous stirring for 15 min to ensure homogenization. The mixtures were spray-applied to the entire fin–plate assembly of a solar still using a gravity-feed spray gun (1.0 mm nozzle, 2.5 bar) in a crosshatch pattern. The average film thickness was 50 ± 5 μm, as verified using a coating gauge.

**Fig. 5:**
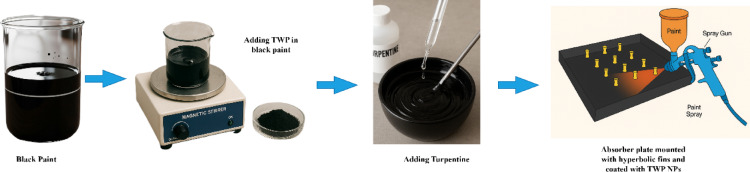
Schematic diagram of step-by-step procedure for fin attached absorber plate coating with TWP NPs.

The coated solar still was cured under ambient conditions (29 ± 2 °C, 55 ± 5% RH) for 24 h, then integrated into a modified single-slope solar still equipped with side-wall mirrors to focus incident solar radiation onto the absorber surfaces. Figure 5 shows the step-by-step procedure of attaching the hyperbolic fins to the absorber plate with the TWP NPs coating.

### Experimental setup

**Fig. 6:**
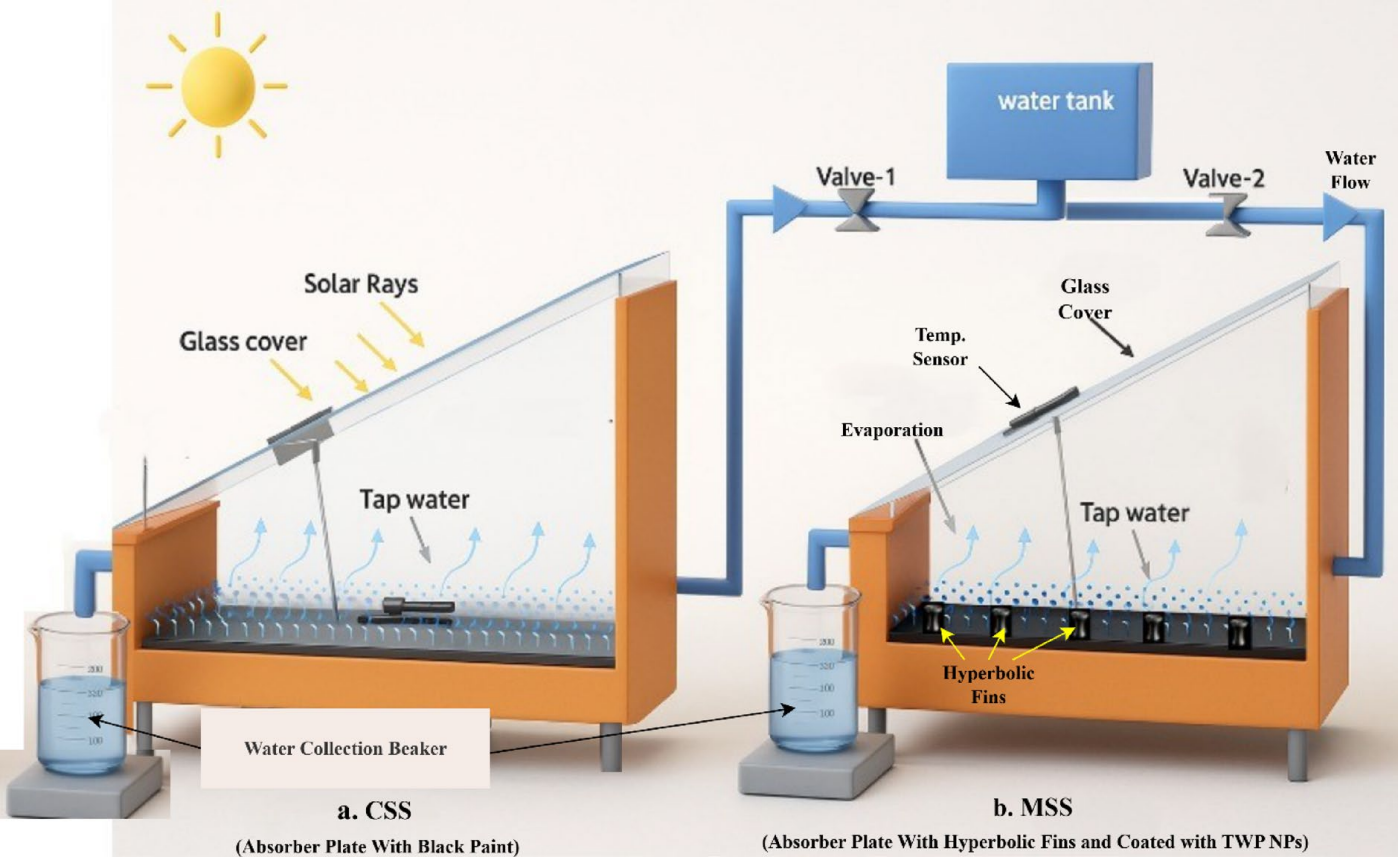
Schematic diagram of CSS and MSS with hyperbolic fins and nanomaterial coating.

**Fig. 7:**
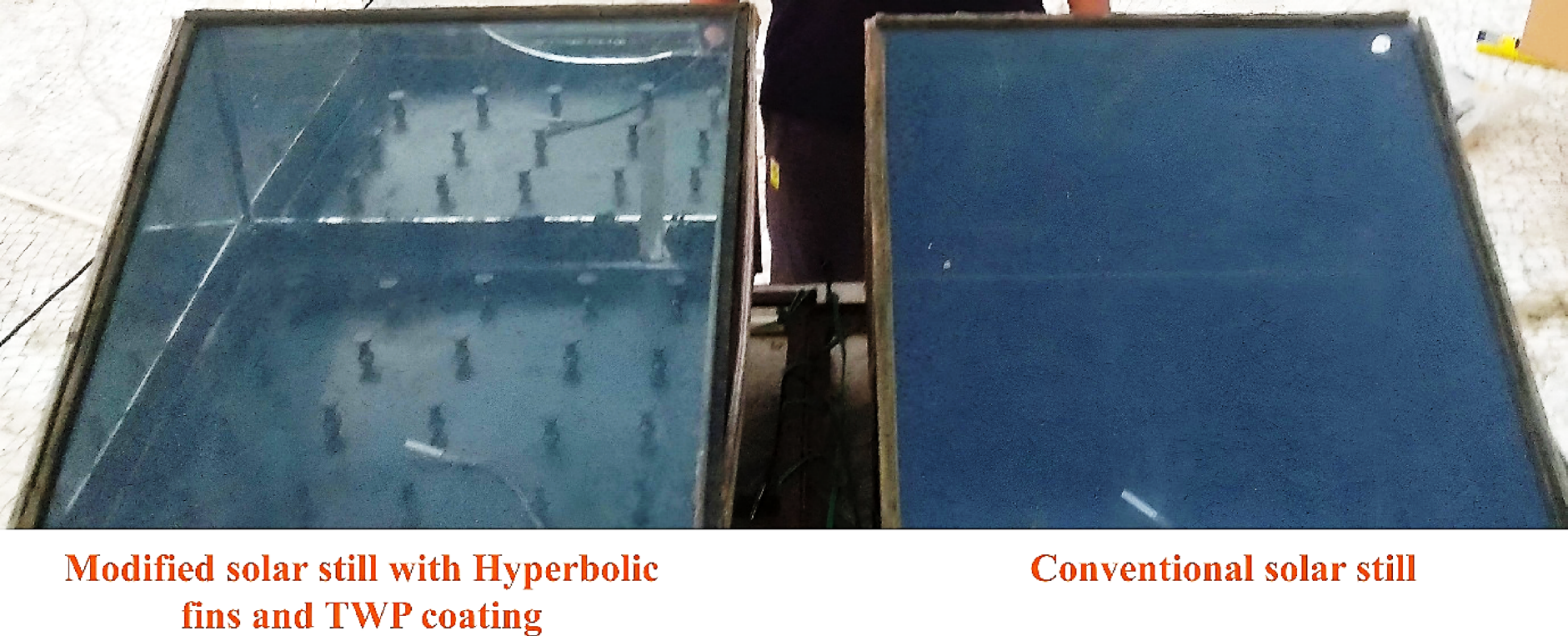
Actual experimental setup of CSS and MSS.

Two identical single-slope solar stills were fabricated to compare the performance of a conventional configuration (CSS) with a modified design incorporating hyperbolic fins and a nanoparticle-enhanced absorber plate (MSS) shown in Figs. 6 and 7.

Each still featured a square basin of 0.50 m × 0.50 m (0.25 m^2^ surface area) made from 2 mm thick galvanised iron (GI) sheet. The side walls were constructed from plywood to minimize thermal losses owing to their insulating properties. The GI basin was shaped with a semicircular channel in its front to guide the condensate to an outlet, which was connected to a collection container via PVC piping. A transparent glass cover, 4 mm thick, was positioned at an inclination matching the site latitude (24.1724° N, 72.4346° E, Palanpur, Gujarat, India). Rubber seals and silicone adhesives were applied to the joints to prevent vapor leakage. Water was supplied from a storage tank through a float-valve mechanism to maintain a constant basin level in both units.

The two systems were tested concurrently under identical climatic conditions in Palanpur between October and December 2023, with daily operation from 07:00 to 19:00 IST. The MSS was evaluated under two operational modes: (i) fins completely submerged in the basin water (3 cm water level) and (ii) fins partially submerged in the basin water (2 cm water level).

To compare the performance of a solar still with a coated hyperbolic fin attached, that is, MSS, to a conventional solar still, various parameters were measured using a different device. The measured parameters included basin water temperature, absorber plate temperature, inner glass surface temperature, ambient temperature, solar irradiance, and distillate production. Temperatures were measured using a DS18B20 waterproof digital thermometer (accuracy ± 0.5 °C), solar radiation intensity measured using an EKO MS-80 S pyranometer (accuracy ± 0.5%), and distillate output measured in laboratory beaker (accuracy ± 2.5). To ensure consistent solar irradiation, all the experiments were conducted under clear-sky conditions. Elminshawy et al.^42^ presented equations for calculating experimental uncertainty, and noted that it should not exceed 4%.

## Results and discussion

To examine the influence of fin geometry and nanoparticle coatings on solar still performance, a conventional solar still was modified by integrating hyperbolic-shaped fins onto the absorber plate and subsequently coating the surface with toner waste powder (TWP) nanoparticles. Experimental trials were conducted in Palanpur, Gujarat (India), between October and December 2023, under natural solar radiation from 07:00 to 19:00 h. Both fully and partially submerged fin configurations were tested to evaluate their effects on basin water heating and distillate productivity. Representative results from November 2023 are presented for subsequent analysis and discussion, as this period offers stable climatic conditions suitable for performance evaluation.

### Ambient temperature and solar radiation intensity trend during the experimental days

**Fig. 8:**
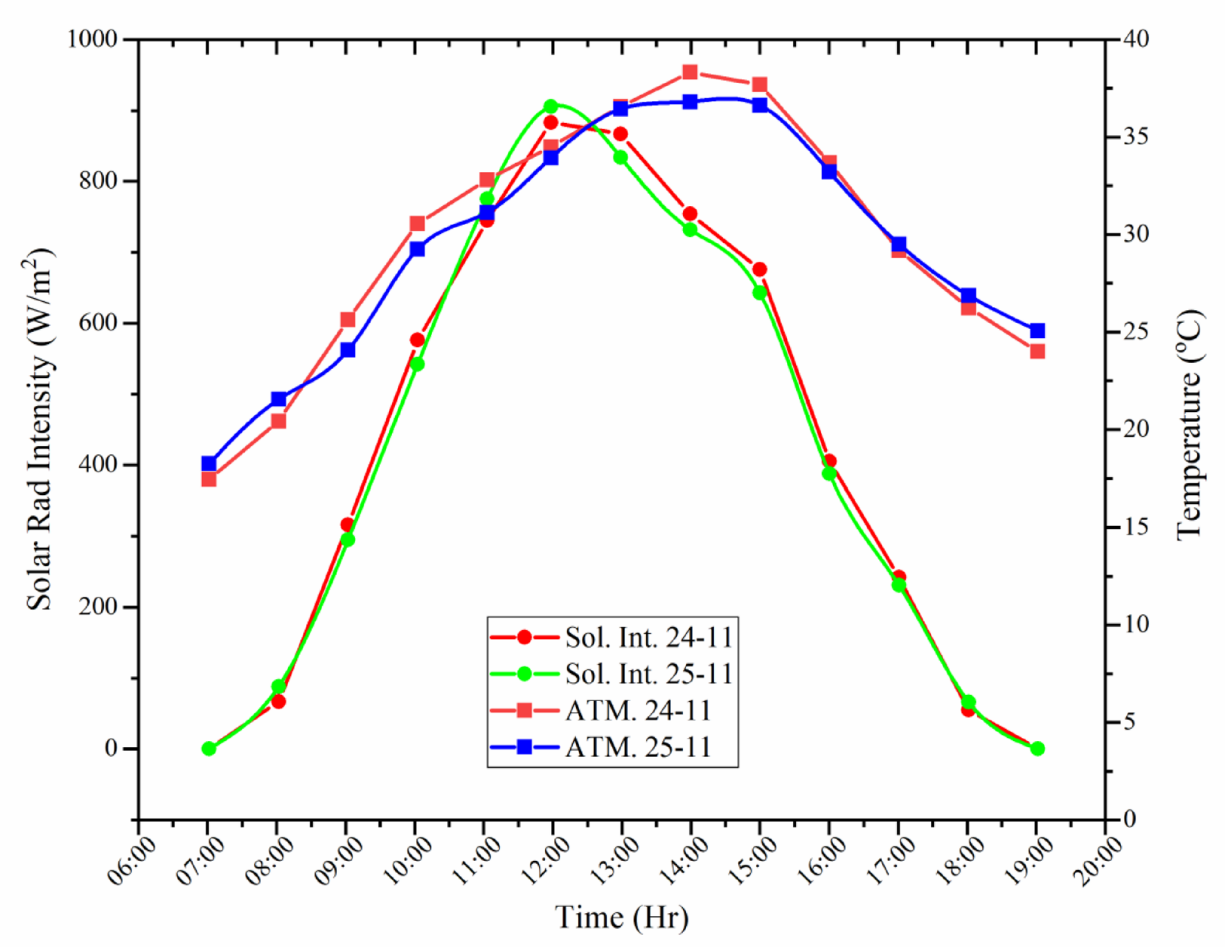
Trend of ambient temp. and solar rad. intensity during experimental days (24th and 25th Nov–23).

The efficiency and performance of solar stills are substantially influenced by the intensity of the solar radiation and ambient temperature. Figure 8 depicts the hourly variation in the solar radiation intensity and ambient temperature recorded on 24th and 25th Nov-23. On both days, solar intensity showed a steep rise after sunrise, reaching maxima of approximately 883 W/m^2^ (24th-Nov) and 905 W/m^2^ (25th-Nov) around midday, and then steadily declined to near-zero by 19:00 h. Ambient temperature followed a comparable trend, but with a noticeable delay, increasing from 18 °C in the early morning to 37–38 °C during early afternoon, before falling to approximately 25 °C in the evening. This delay arises from the thermal inertia of the ground-air system, where absorbed solar energy is stored on the surface and gradually transferred to the atmosphere by conduction and natural convection. The similarity in profiles for the two days suggests predominantly clear sky conditions and minimal atmospheric attenuation. For solar still operation, such conditions are favorable: strong irradiance enhances basin heating and vapor generation, whereas elevated ambient temperatures reduce convective losses to the surroundings. The observed lag between radiation and air temperature also implies that the maximum evaporation potential is determined by the coupled dynamics of radiative input and environmental heat storage rather than instantaneous solar intensity alone.

### Water temperature variations of CSS and MSS for partially and fully submerged fins

**Fig. 9:**
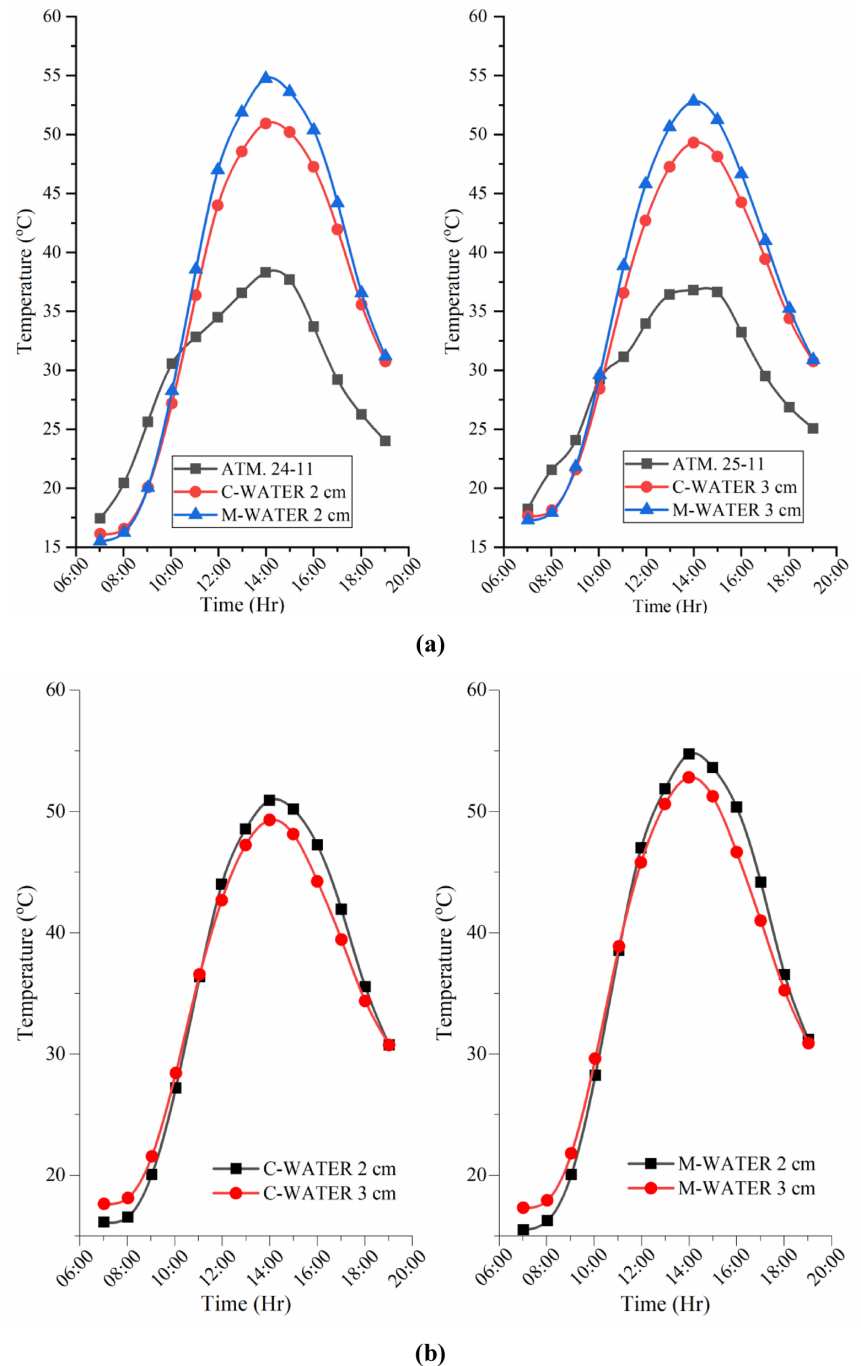

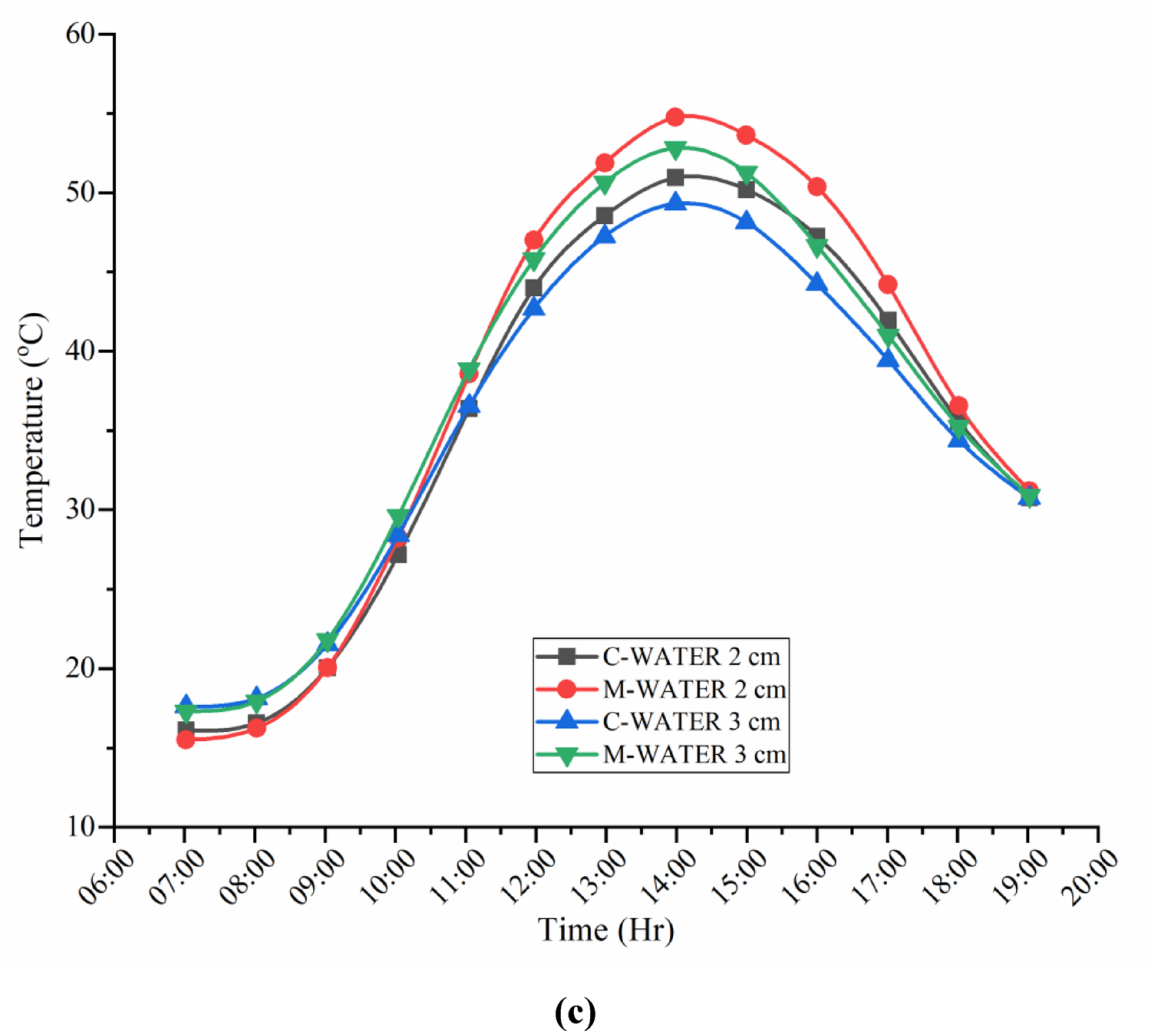
(**a**) Water temperature variations during the experimental days. (**b**) Water temperature variations of CSS and MSS for partially and fully submerged fins. (**c**) Water temp. variations of CSS and MSS during the experimental days.

Figure 9a–c show the hourly profiles of basin water and ambient temperatures for the conventional and modified solar stills with partially and fully submerged fins. In both configurations, the water temperature increased with the ambient temperature, reaching its highest value at approximately 14:00. Peak basin temperatures for the conventional still were 50.94 °C (partially submerged) and 49.31 °C (fully submerged), whereas the modified unit with hyperbolic fins and a TWP-coated absorber achieved 54.75 °C and 52.81 °C, respectively. The modified still with hyperbolic fins and a TWP-coated absorber achieved ~ 4 °C higher basin temperatures than the conventional design. This enhancement arises from the increased absorber-water contact area provided by the fins, which improves conductive heat transfer, and from the nanocoating, which boosts the solar absorptivity. Even a modest temperature rise substantially elevates the vapor pressure at the water surface, thereby strengthening the driving force for evaporation and improving the distillate yield.

**Fig. 10:**
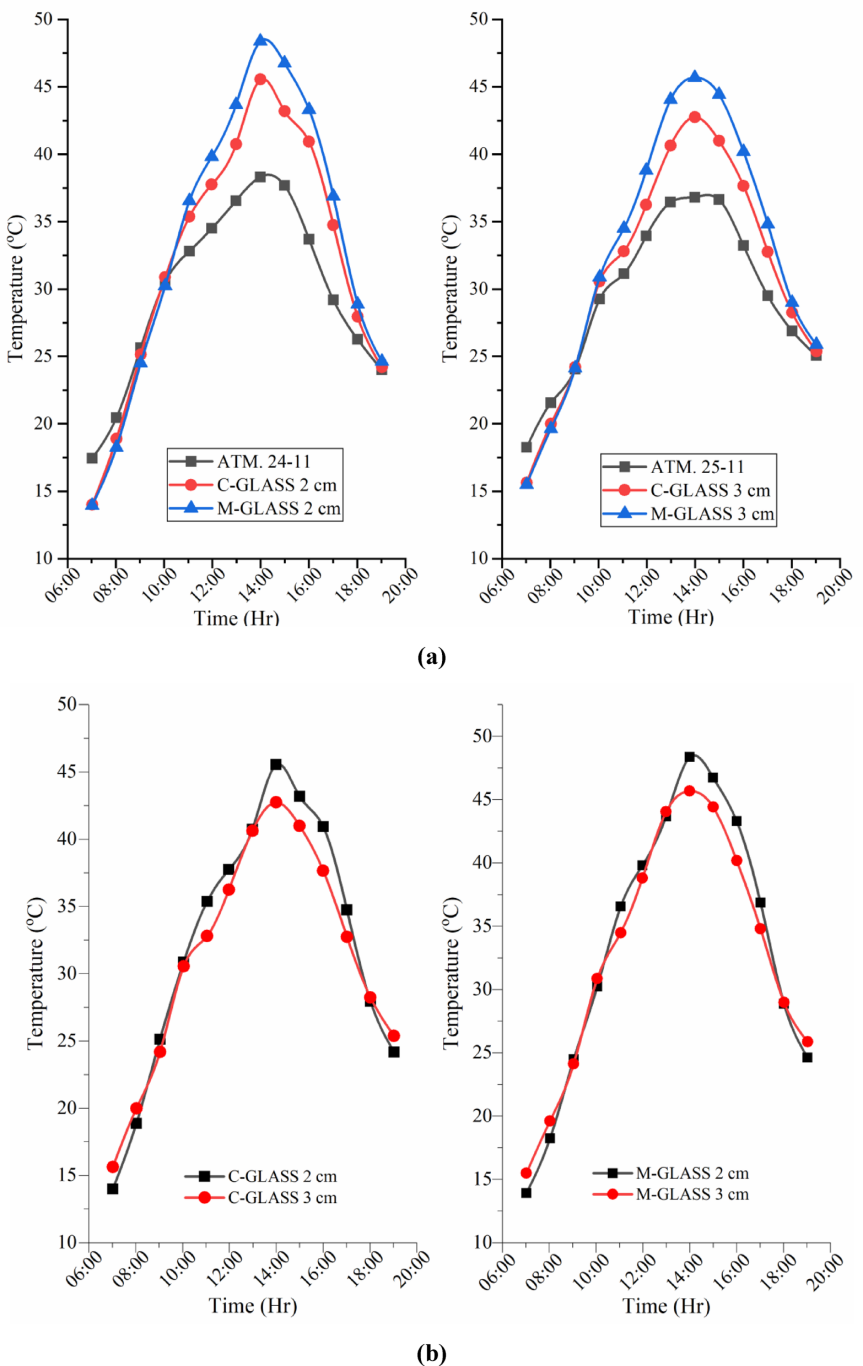

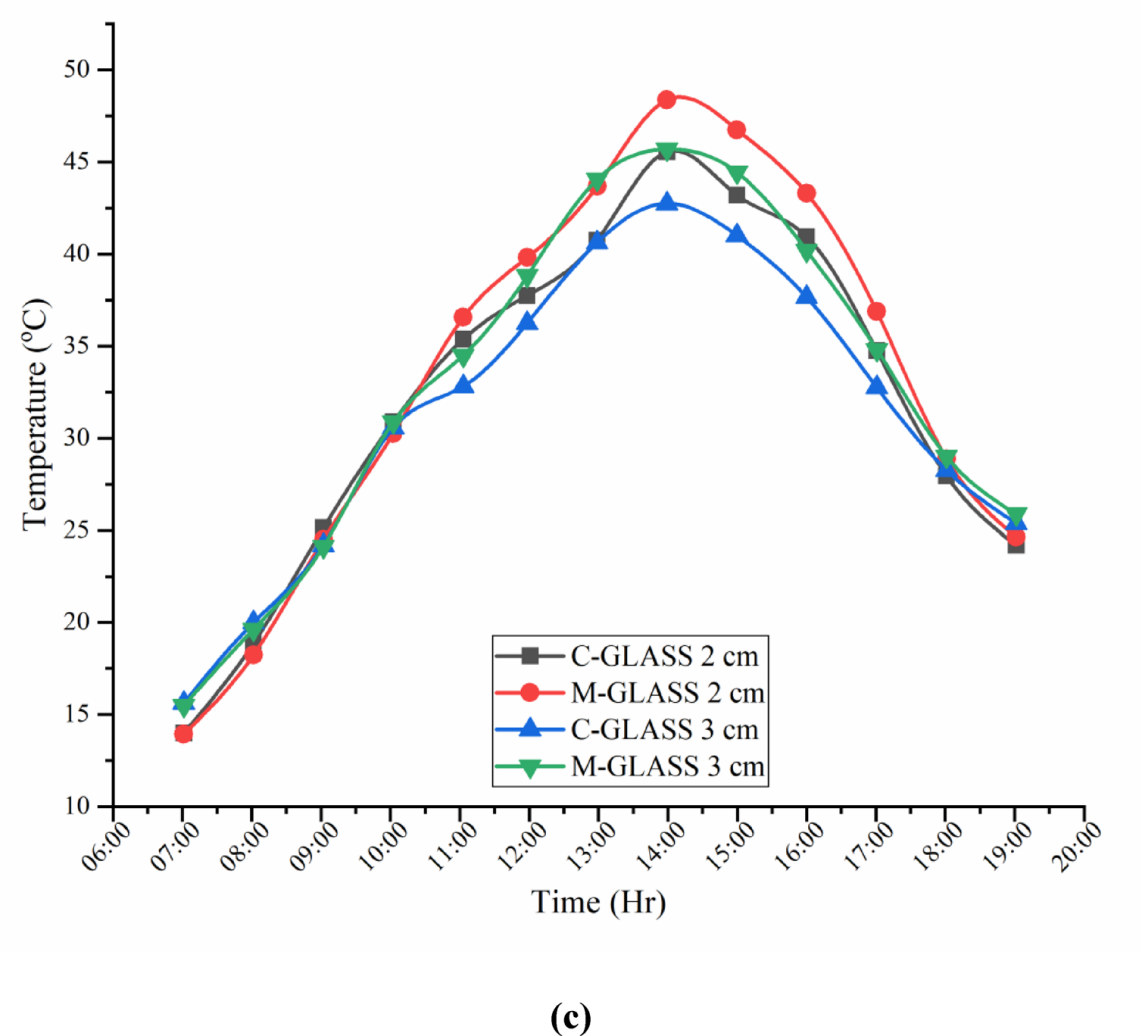
(**a**) Glass surface temperature variations during the experimental days. (**b**) Glass surface temperature variations of CSS and SSWCHF (MSS) for partially and fully submerged fins. (**c**) Glass surface temperature variations during the experimental days.

**Fig. 11:**
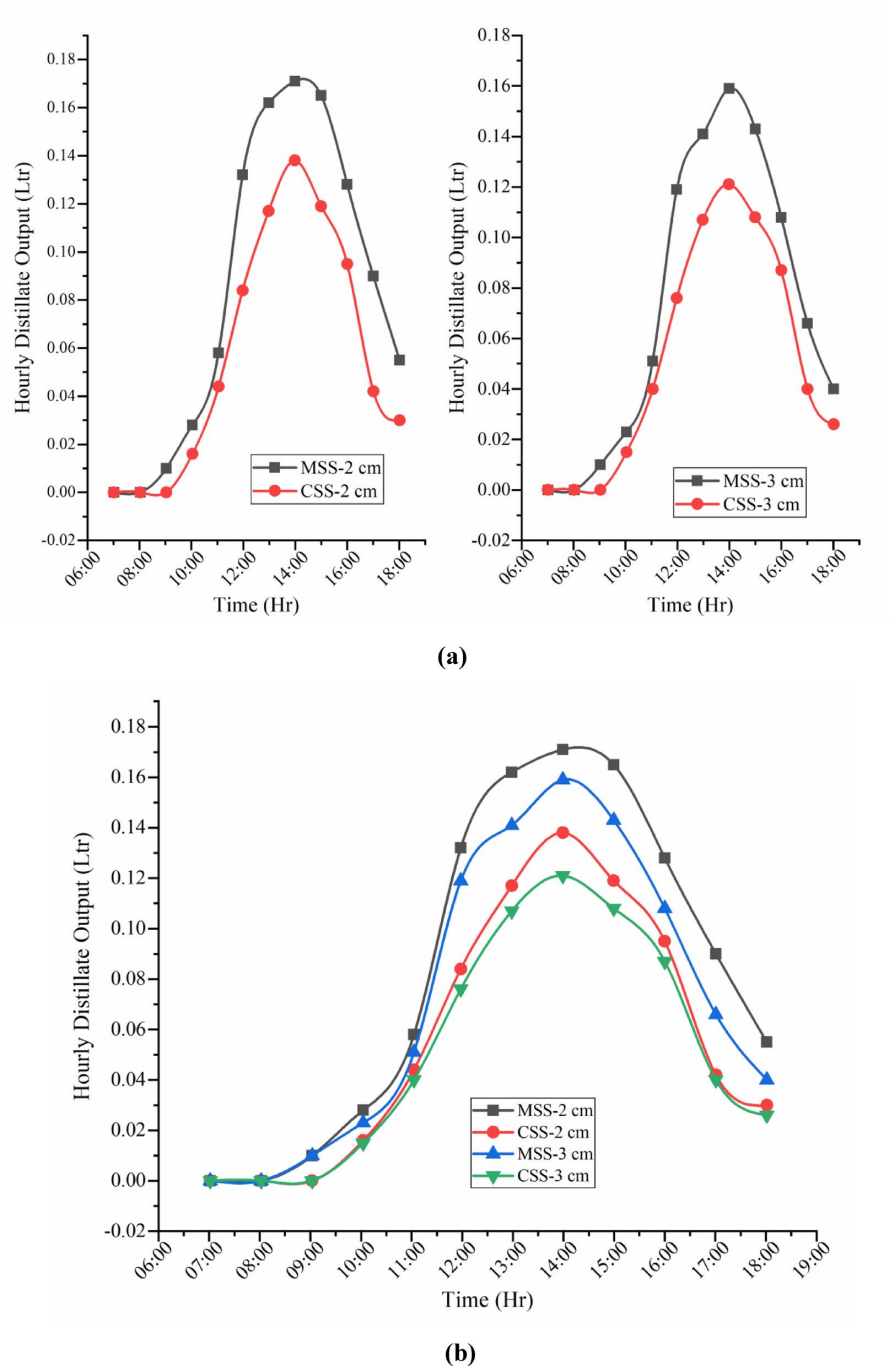
(**a**) Hourly distillate production of CSS and MSS under partially (2 cm) and fully (3 cm) submerged fin configurations. (**b**) Hourly distillate production of CSS and MSS for partially and fully submerged fins.

### Glass surface temperature variatioons of CSS and MSS for partially and fully submerged fins

Figure 10a–c present the hourly variation of transparent glass cover temperature for CSS and MSS partially and fully submerged fin conditions. In both designs, cover temperature followed ambient temperature trends, remaining lower during morning and evening hours and peaking at 14:00 h. The CSS recorded maximum of 45.56 °C (2 cm water level) and 42.75 °C (3 cm water level), while the MSS unit reached 48.38 °C (partially submerged) and 45.69 °C (fully submerged), respectively. The modified still exhibited slightly higher cover temperatures than the conventional unit, with partially submerged fins producing greater values than fully submerged fins due to more effective basin heating and stronger convective flux. However, the differences between designs were smaller than those observed for basin water, indicating that glass cover heating is largely an indirect response to basin temperature rather than a direct effect of absorber modifications.

### Distillate production of CSS and modified SSWCHF (MSS)

**Fig. 12:**
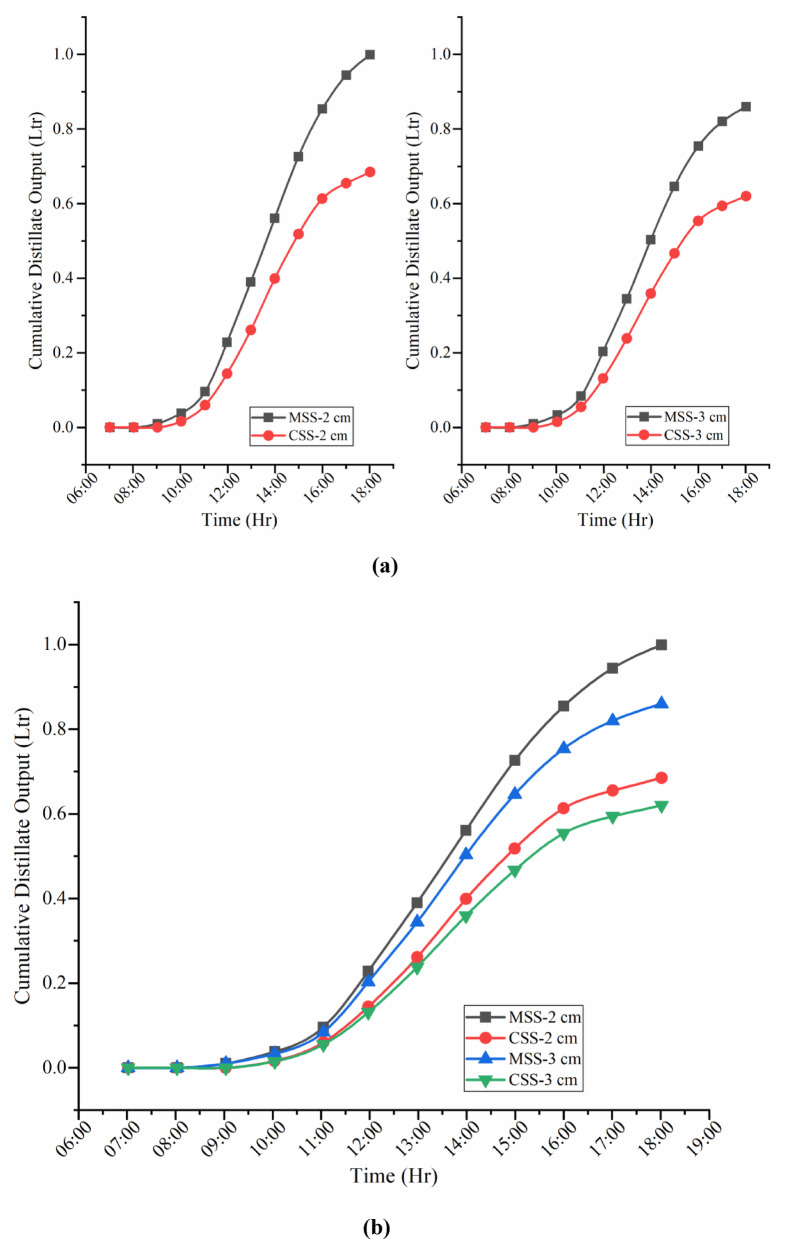
(**a**) Cumulative distillate production of CSS and MSS under partially (2 cm) and fully (3 cm) submerged fin configurations. (**b**) Cumulative distillate output of CSS and MSS.

Figure 11a,b and 12a,b present the comparative hourly and cumulative distillate performance of CSS and MSS under partially (2 cm) and fully (3 cm) submerged fin configurations. Hourly profiles closely followed the diurnal solar radiation pattern, with negligible deviations in early morning and evening but substantial divergence between 10:00–18:00 h. Peak productivity occurred at 14:00 h, where the modified system achieved 159 ml (partial) and 121 ml (full) compared to the conventional still’s 171 ml and 138 ml, respectively, for a 0.25 m^2^ basin.

Cumulative production rose steadily throughout the day, with the steepest growth occurring between 10:00 and 14:00. The modified SSWCHF attained 0.999 L (partial) and 0.860 L (full), surpassing the conventional still’s 0.685 L and 0.620 L, respectively, corresponding to a 45.84% overall enhancement.

The performance gains are attributed to the synergistic effect of hyperbolic fins, which increase the effective heat transfer surface area and strengthen conductive heat transfer and thermal distribution, and toner waste powder (TWP) nanomaterial coatings, which elevate solar absorptivity while suppressing thermal losses. Together, these modifications raise the basin water temperature, intensify the vapor pressure gradient across the water–glass interface, and sustain evaporation beyond sunset owing to delayed cooling. Thus, the performance enhancement is not only a function of peak midday heating but also reflects a more efficient utilization of the daily solar energy input.

Partially submerged fins still enhance the performance by offering a larger effective contact area for heat transfer while maintaining a lower thermal inertia, allowing absorbed solar energy to be transferred to the basin water more rapidly. Fully submerged fins, although providing a greater wetted surface area, introduce additional thermal mass, which stores part of the absorbed heat and delays its transfer to the water. This higher inertia limits the rate of temperature rise during peak solar hours, resulting in lower evaporation rates compared with the partially submerged configuration.

### Comparative assessment of distillate yield with the literature

The analysis shows that nanomaterials like Al₂O₃ and CuO nanoparticles can boost productivity by up to 38% and 30% through improved thermal conductivity, while TiO₂ offers only a modest 6% gain. Biomass additives, such as pistachio shell powder, performed even better, with a 46.26% improvement, highlighting their eco-friendly potential. Structurally, plate-type fins increased output by 15.5%, whereas hollow copper fins combined with PCM tanks achieved over 43% with fins alone, and more than 100% with PCM integration. Similarly, PCM with pin fins delivered a 30% boost, underlining the strong impact of geometry-storage synergy. In this study, hyperbolic fins enhanced with toner waste powder achieved a 45.84% improvement-comparable to top-performing nanoparticle and PCM systems. This approach stands out for combining an innovative fin design with low-cost waste materials, offering a sustainable and cost-effective alternative to conventional nanomaterials and metals (Table 2).

**Table 2:** Comparative assessment of distillate yield with the literature.

Reference	Material/modification	Reported distillate improvement (%)	Remarks
Kumar et al.^8^	Al_2_O_3_ nanoparticles on the absorber plate	~ 38%	At 1 cm water depth: 3.48 L, 38.65% efficiency; productivity ↑ 38.09%
Gupta et al.^9^	0.12% CuO in water, depths 5 cm and 10 cm	~ 22.4 to 30%	Productivity ↑ 22.4% & 30%.
Kabeel et al.^11^	TiO_2_ coated absorber in pyramid still	~ 6.1%	Improved water temperature and distillate output.
Noman et al.^24^	Pistachio shell powder	46.26%	Improved distillate output.
Panomwan et al.^26^	Plate-type fins	15.5%	Plate type fins attached in standard solar still.
Kateshia et al.^32^	PCM and pin fins	30%	With PCM and fins improvement is 30% than only fins.
Kabeel et al.^28^	Hollow copper circular fins	43%	Improvement with copper fins.
Present study	Hyperbolic fins + Toner Waste Powder	45.84%	Comparable to metallic NPs, PCM and fins, a cost-effective

### Thermal analysis (energy, exergy efficiency)

Thermodynamic analysis was carried out to assess the performance of the conventional solar still (CSS) and the modified still with hyperbolic fins and TWP nanocoating (MSS). In the present study, a thermal performance evaluation was conducted for both CSS and MSS with partially submerged fins, as this configuration demonstrated a superior distillate yield compared to fully submerged fins. The analysis combined both energy and exergy perspectives to capture not only the magnitude of useful energy conversion but also the quality of that energy in driving evaporation.

### Energy-exergy analysis of SSWCHF and CSS

**Fig. 13:**
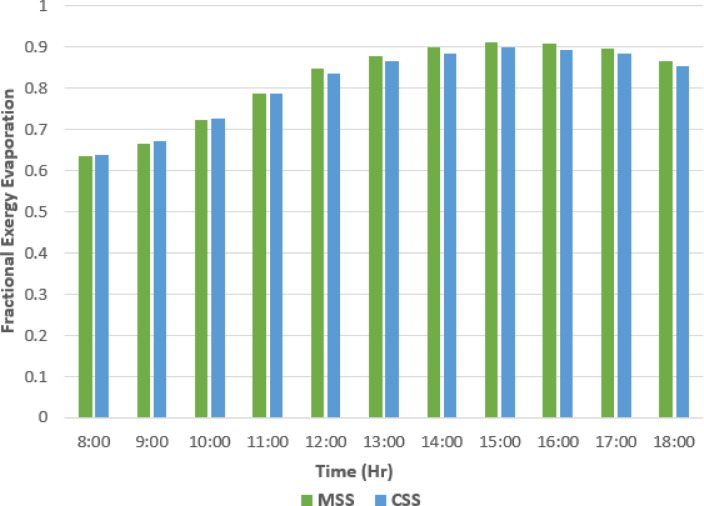
Fractional exergy evaporation variation with time.

**Fig. 14:**
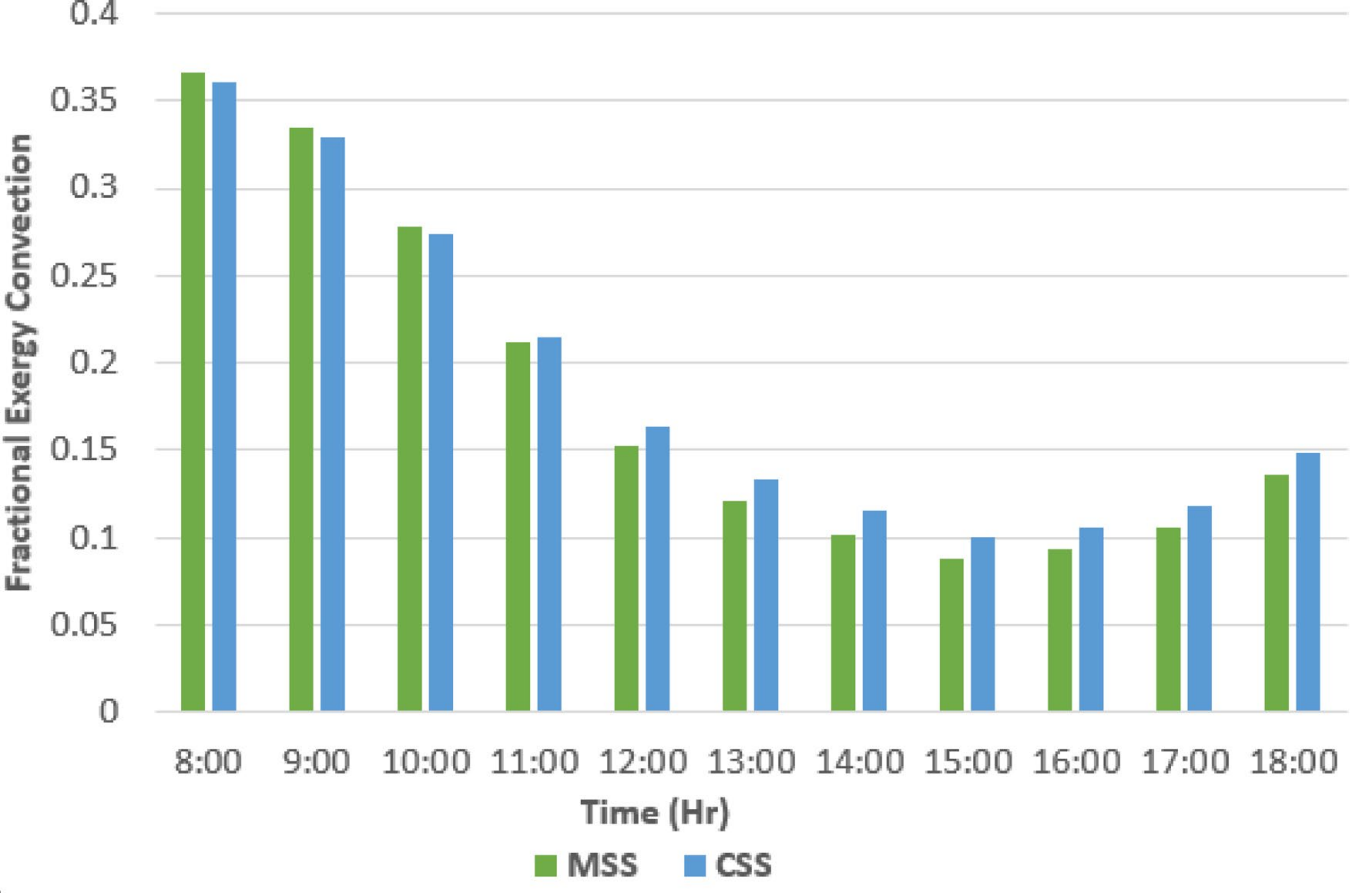
Fractional exergy convection variation with time.

**Fig. 15:**
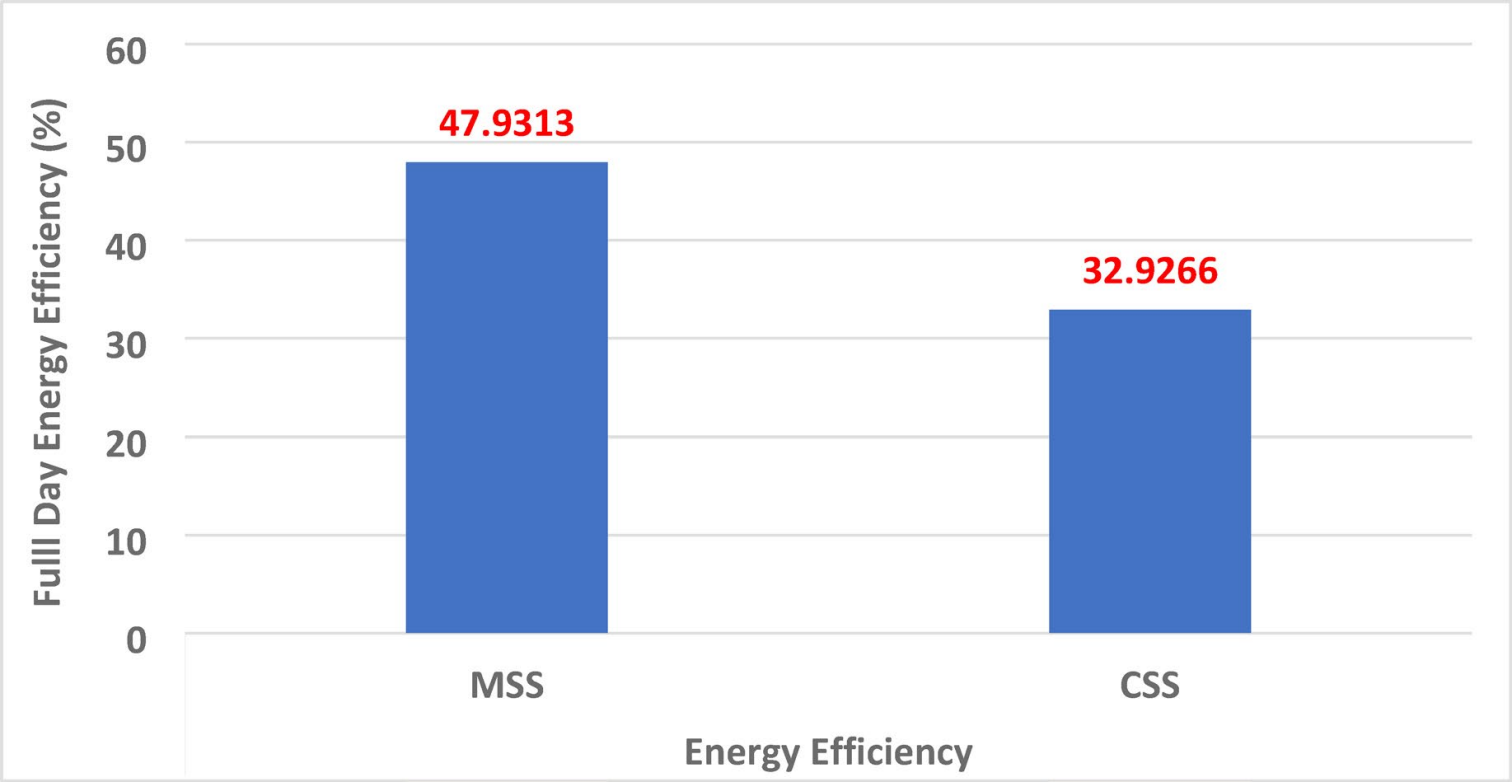
Energy efficiency variations of MSS and CSS.

**Fig. 16:**
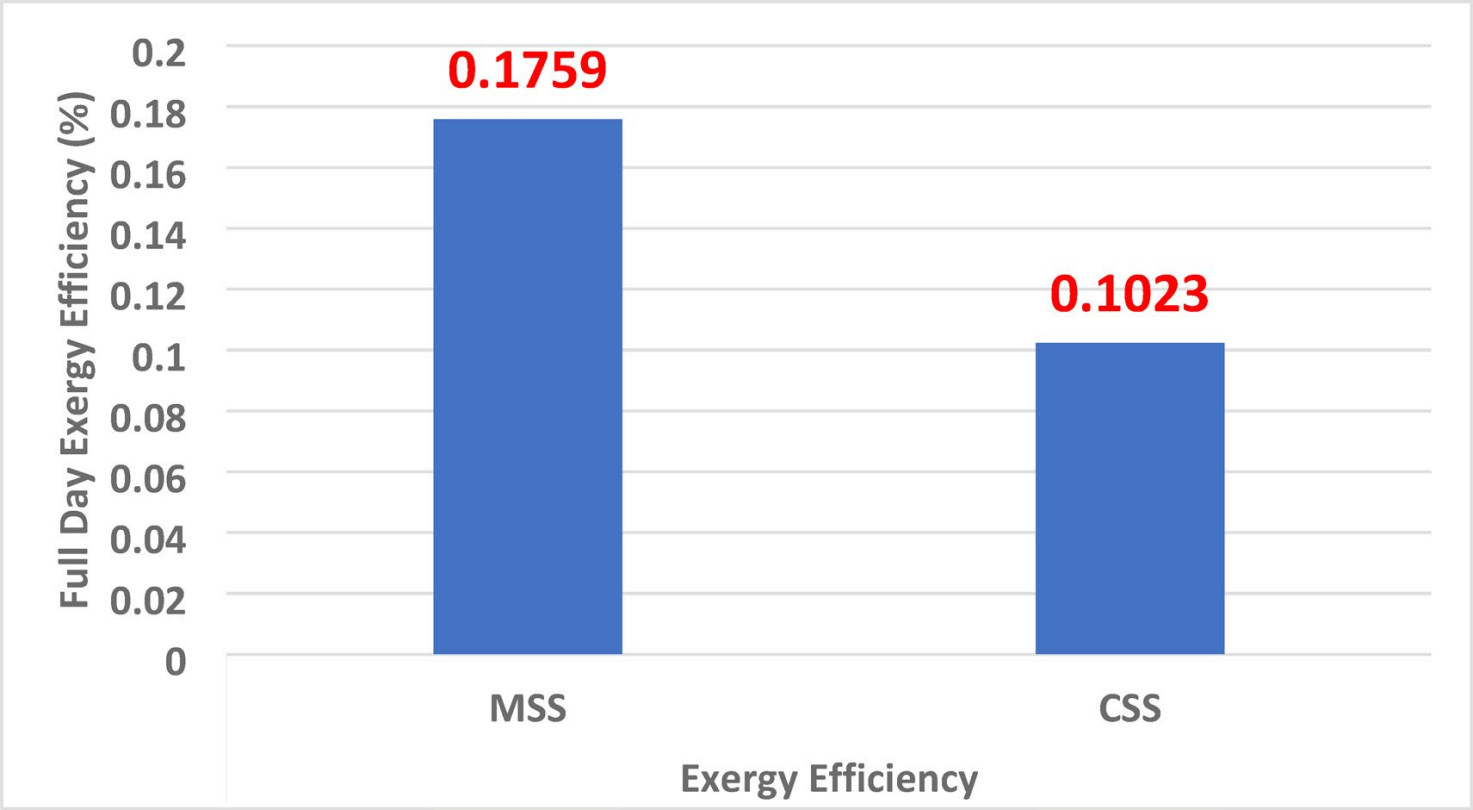
Exergy efficiency variation of MSS and CSS.

Figures 13, 14, 15 and 16 present the comparative performance of the two stills, showing the effect of absorber surface modifications on fractional exergy distribution and overall efficiency. The integration of fins and nanomaterial coatings directly influences heat transfer pathways, altering the partition between evaporation and convection and thereby affecting both energy yield and exergy utilization.

### Fractional exergy variations for evaporation (*F*_*e, bw-tg*_)

As shown in Fig. 13, the evaporative exergy fraction increased progressively from morning to early afternoon, reaching its maximum during the peak solar irradiance before declining slightly toward the evening. The MSS consistently exhibited higher values (0.63–0.91) than the CSS because of the elevated basin water temperatures achieved through improved solar absorption and fin-induced heat transfer. Mechanistically, the fins enhance the thermal coupling between the absorber and basin water, while the TWP coating improves optical absorption and reduces radiation losses. Together, these effects increase the evaporative heat transfer coefficient, directing a larger share of the available exergy to the phase change. The fractional exergy for evaporation (*F*_*e, bw−ig*_) was calculated using Eq. (1)^14^.

1 $$\:{F}_{e,bw-ig}=\frac{{Ex}_{e,bw-ig}}{{Ex}_{ti}}$$

where $\:{Ex}_{e,bw-ig}$ evaporative exergy and $\:{Ex}_{ti}$ are the total heat transfer calculated using the following equations^43^:

$$\:{Ex}_{e,bw-ig}={h}_{e,bw-ig}\times\:{A}_{bw}\:\times\:\left({T}_{bw}-{T}_{ig}\right)\times\:\left(1-\frac{{T}_{a}}{{T}_{bw}}\:\right)$$

$$\:{Ex}_{ti}={h}_{t}\times\:{A}_{g}\times\:\left({T}_{bw}-{T}_{ig}\right)\left(1-\frac{{T}_{a}}{{T}_{bw}}\right)$$

#### Fractional exergy variations for convection (*F*_*c, bw-ig*_)

Figure 14 shows that the fractional convective exergy decreased from morning to midday, with values dropping from ~ 0.36 at 08:00 h to ~ 0.08 during peak radiation, and recovering slightly in the late afternoon. The MSS recorded marginally lower convective fractions because its higher water temperature favored evaporation over sensible heat loss. In effect, the system reallocates thermal exergy to the latent processes, which are more productive for distillation. The fractional exergy for convection (*F*_*c, bw-ig*_) was calculated using Eq. (2)^14^.

2 $$\:{F}_{c,bw-ig}=\frac{{Ex}_{c,bw-ig}}{{Ex}_{ti}}$$

where, $\:{Ex}_{c,bw-ig}$ convective exergy calculated using the following equations^14^.

$$\:{Ex}_{c,bw-ig}={h}_{c,bw-ig}\times\:{A}_{bw}\times\:\left({T}_{bw}-{T}_{ig}\right)\times\:\left(1-\frac{{T}_{a}}{{T}_{bw}}\:\right)$$

### Energy efficiency variations (η_*energy*_)

Figure 15 indicates that CSS achieved an energy efficiency of 32.93%, whereas MSS reached 47.93%, corresponding to a 45.6% improvement. This gain reflects increased freshwater yield, more efficient transfer of heat from the basin to the cover, and reduced reflective losses due to the nanomaterial coating. The efficiency is determined as^43^

3 $$\:{\eta\:}_{D}=\frac{\varSigma\:{m}_{D}\times\:L}{\varSigma\:{A}_{g}\times\:{I}_{t}}$$

where $\:{m}_{D}$ is the daily freshwater mass (kg), *L* is the latent heat of evaporation (J/kg), and *A*_*g*_ is the glass cover area (m^2^), and *I*_*t*_ is the total solar radiation (W/m^2^).

### Exergy efficiency variations (η_*exergy*_)

Figure 16 shows exergy efficiencies of 0.1023% for CSS and 0.1759% for MSS, corresponding to a 71.99% relative improvement. The higher performance of the MSS is attributed to enhanced evaporative exergy and reduced convective losses, which together increase the proportion of absorbed solar energy converted into useful work potential. The exergy efficiency was evaluated as follows^44^:

4 $$\:{\eta\:}_{exe}=\frac{{Ex}_{out}}{{Ex}_{in}}$$

where, $\:{Ex}_{out}\:$= Exergy output value, which is equal to the evaporation of exergy between the basin water and the inner glass cover ($\:{Ex}_{e,bw-ig}$). $\:{Ex}_{in}$ = Exergy input value; the exergy input value equals the absorbed solar radiation, calculated using the following equation:

$$\:\:\:{Ex}_{in}={A}_{bw}\times\:{I}_{t}\times\:\left[\:1-\frac{4}{3}\:\times\:\:\left(\frac{{T}_{a}+273.15}{Ts}\right)\:+\frac{1}{3}\:\times\:{\left(\frac{{T}_{a}+273.15}{Ts}\right)}^{4}\right]$$

The results confirm that the MSS is thermodynamically superior, not only increasing the total energy utilization, but also improving the quality of that energy by channeling a greater share into evaporation rather than convection. The combined action of hyperbolic fins and nanomaterial coating elevates the basin temperature, strengthens the vapor pressure gradients, and sustains evaporation even during declining irradiance, making the system more effective both energetically and exergetically.

### Statistical analysis

A two-tailed Student’s t-test was performed on the daily measurements of energy and exergy efficiencies to compare the CSS and MSS. As shown in Table 3, MSS achieved significantly higher energy (47.93% vs. 32.93%) and exergy efficiencies (0.1759 vs. 0.1023), with both cases yielding t-values above 7.5 and *p* < 0.001. These results confirm that the observed improvements are statistically significant and directly attributable to system modifications.

**Table 3:** Statistical comparison of CSS and SSWCHF (MSS) performance metrics.

Metric	Mean (CSS)	Mean (MSS)	t-value	*p*-value	Significance
Energy efficiency (%)	32.93	47.93	8.21	< 0.001	Significant
Exergy efficiency (%)	0.1023	0.1759	7.54	< 0.001	Significant

### Economic analysis of CSS and MSS

The economic performance was assessed using a cost–benefit approach that considered the manufacturing cost, primary annual charge (PAC), distillate yield, and unit water production cost (Table 4). The MSS incurred a higher fabrication cost (USD 28.15) relative to CSS (USD 20.65), primarily due to the addition of hyperbolic fins and side mirrors, which increased capital expenditure by ~ 36%. This translated to a PAC of USD 4.20 for MSS, compared to USD 3.08 for CSS.

Despite a higher initial investment, the MSS consistently produced higher freshwater output (0.999 L/day; 299.7 L/year per 0.25 m^2^) compared to CSS (0.685 L/day; 205.5 L/year). Mechanistically, the productivity gain arises from the enhanced heat transfer surfaces and improved optical absorption, which collectively sustain higher basin water temperatures and longer evaporation hours. Consequently, the unit cost of water dropped from USD 0.0158/L (CSS) to USD 0.0148/L (MSS). This marginally lower production cost, combined with improved reliability and yield, confirms that additional capital input is economically justified. Such trends are consistent with previous studies reporting that thermal augmentation strategies increase the financial viability of solar desalination systems by improving the return per unit investment.

**Table 4:** Cost analysis of CSS and SSWCHF.

Sr.	Instrument	CSS	MSS
		(USD ֆ)	(USD ֆ)
1	Absorber surface area of G.I. Sheet	2.5	2.5
2	Insulating material and plywood	5.4	5.4
3	Transparent glass cover	3	3
4	Paint of black colour	2.25	2.25
5	Distilled water collector	3.125	3.125
6	Sealant	1.25	1.875
7	Side mirror	–	3.75
8	Other	3.125	3.125
9	Hyperbolic fins	–	2.5
9	Total cost	20.65	28.15
10	Primary annual charge (PAC)	3.0775	4.1952
11	Full day distillate (L/0.25 m^2^)	0.685	0.999
12	Yearly distilled water generation (L/0.25 m^2^)	205.5	299.7
13	Cost of water/L (USD ֆ)	0.0158	0.0148

### Exergo-environmental analysis of CSS and MSS

The exergo-environmental performance was quantified using the annual exergy generation and carbon credit production (CCP) metrics (Table 5). The MSS demonstrated a higher annual exergy generation, consistent with its improved thermodynamic performance, resulting in greater CO_2_ emission offsets when compared to the CSS. Carbon credits were calculated using Eqs. (5) and (6)^45^.

5 $$\:CCP=\:\varphi\:{ex,co}_{2}\times\:Zc{o}_{2}$$

6 $$\:\varphi\:{ex,co}_{2}=\:\frac{\left(Exout\:\times\:l\right)\times\:2}{1000}$$

where $\:\:\varphi\:{ex,co}_{2}$is the exergo-environmental value, $\:{Ex}_{out}$ is the exergy output, *l* is the life of the solar still, and $\:Zc{o}_{2}$ is the average price of carbon = 14.5 USD.

Mechanistically, the gains stem from the improved exergy distribution within the MSS, where fins and nanocoating suppress convective losses and channel a greater share of absorbed energy into the high-grade evaporative exergy. This thermodynamic optimization enhances both the water productivity and CO_2_ mitigation potential. The reduced net payback time (3.76 months for MSS vs. 4.02 months for CSS) underscores the dual advantage of faster capital recovery and lower environmental footprint.

**Table 5:** Environmental analysis of CSS and MSS.

S.*N*.	Parameters	CSS	MSS
1	Yearly total life of SS in years	10	10
2	Yearly distillate yield (L/0.25m^2^)	205.5	299.7
3	Primary Annual Charges PAC (In USD)	3.0775	4.1952
4	Output Exergy Ex_out_, (W)	0.1106	0.1901
5	Exergo-economic Parameter Rex (W/USD)	0.0359	0.0453
6	Exergo-environmental Parameter ϕ_ex_, _CO2_ (t CO_2_/year)	0.0022	0.0038
7	Carbon credit produced CCP (USD/year)	0.0321	0.0551
8	Net pay back time (NPBT) in Month (Days)	4.02	3.76

### Integrated thermo-economic-environmental performance

Table 6 lists the thermodynamic, economic, and environmental indicators. The MSS showed marked improvements in energy efficiency (+ 45.6%), exergy efficiency (+ 72%), and annual distillate yield (+ 46%), alongside reduced unit water cost and substantially greater carbon credit potential (+ 71.6%). These synergistic benefits highlight that the design modifications, hyperbolic fins, and TWP nanocoating do not merely increase the thermal performance but also enhance the financial viability and environmental sustainability.

**Table 6:** Thermo-economic-environmental performance of CSS and MSS.

Parameter	CSS	MSS	Improvement (%)
Fractional evaporative exergy (Max)	0.89	0.91	+ 2.25
Fractional convective exergy (Max)	0.36	0.35	− 2.78
Energy efficiency (%)	32.93	47.93	+ 45.57
Exergy efficiency (%)	0.1023	0.1759	+ 71.97
Daily distillate yield (L/0.25 m^2^)	0.685	0.999	+ 45.9
Yearly distillate yield (L/0.25 m^2^)	205.5	299.7	+ 45.9
Unit water cost (USD/L)	0.0158	0.0148	− 6.33
Carbon credit potential (USD/year)	0.0321	0.0551	+ 71.6

**Fig. 17:**
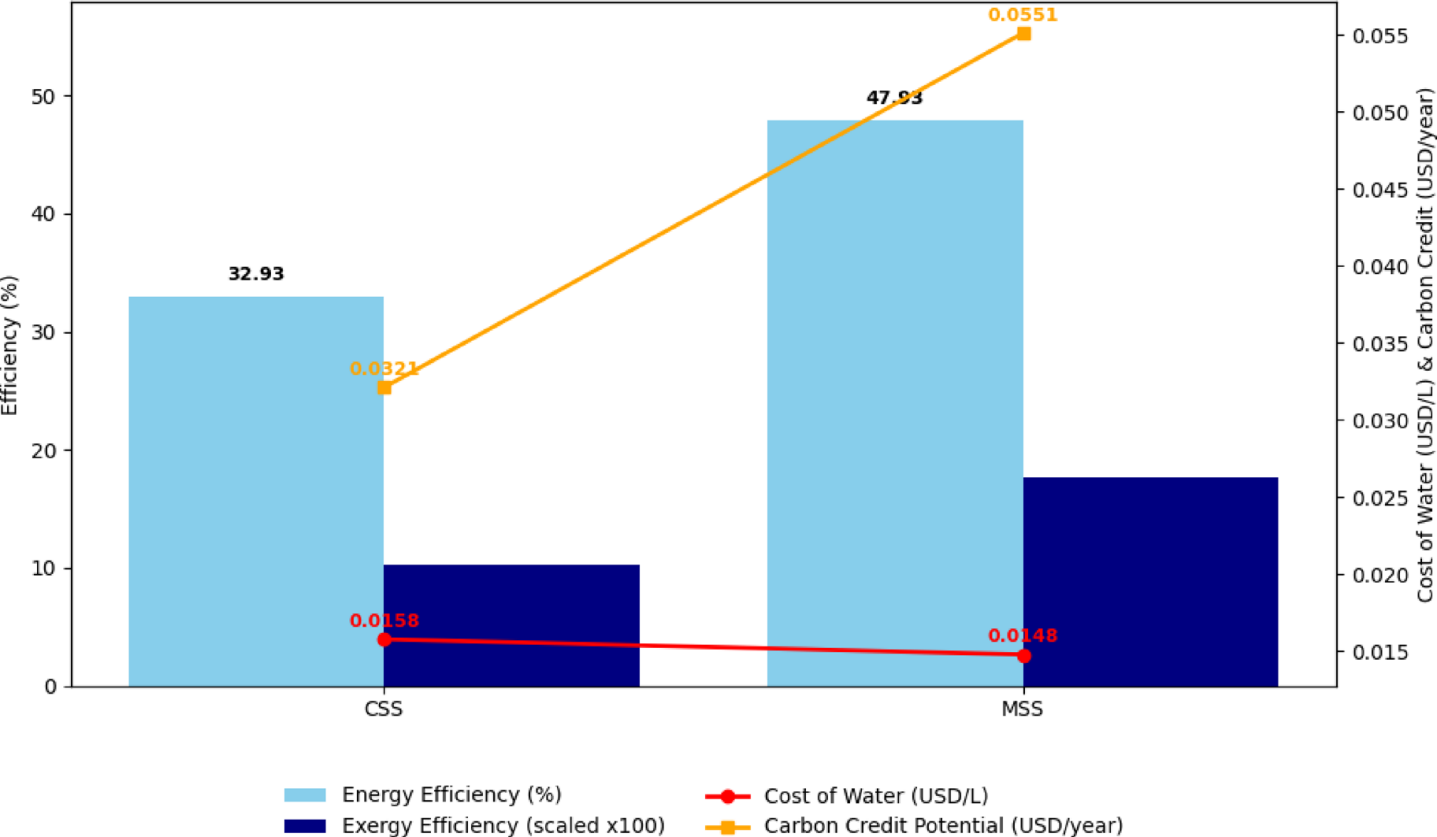
Integrated performance of CSS and MSS (thermodynamic, economic and environmental performance).

Figure 17 presents Overall, the MSS presents a mechanistically optimized desalination device: fins increase the effective surface area for heat transfer, nanocoating elevates absorptivity while suppressing re-radiation losses, and both modifications improve exergy allocation toward evaporation. These mechanisms collectively enable superior water productivity, faster economic returns, and greater CO_2_ offset potential, positioning MSS as a robust and sustainable desalination technology for deployment in water-scarce, off-grid, and sustainability-driven contexts.

## Conclusions

In this study, a modified single-slope solar still with composite hyperbolic fins (MSS) integrated with toner waste powder (TWP) nanocoating and side mirrors was developed for thermodynamic, economic, and environmental optimization over a conventional solar still (CSS). The novelty lies in coupling geometric fin modification with nanomaterial-enhanced absorption to simultaneously boost the energy/exergy efficiency, freshwater yield, and sustainability performance. A comparative experimental evaluation was conducted between the CSS and MSS under identical meteorological conditions. The performance was assessed through basin and cover temperature measurements, distillate yield, thermal (energy/exergy) analysis, and techno-economic-environmental metrics. Both partially and fully submerged fin configurations were tested, with the partially submerged case showing a superior response owing to the lower thermal inertia. The major findings are summarized as follows:

- Basin water temperature increased by ~ 4 °C in MSS compared to CSS, peaking at 54.8 °C (vs. 50.9 °C).
- Daily distillate yield improved by 45.84% (0.999 L vs. 0.685 L per 0.25 m²), with the maximum hourly output recorded from 2:00–3:00 pm.
- Energy efficiency increased from 32.9% (CSS) to 47.9% (MSS), a 45.6% enhancement.
- Exergy efficiency increased from 0.102% to 0.176%, a **~** 72% relative gain.
- Unit water cost decreased from USD 0.0158/L to 0.0148/L, despite a 36% higher manufacturing cost.
- Exergo-environmental benefits include an increase in CO₂ offset from 0.0022 to 0.0038 t/year, corresponding to a 71.6% increase in carbon credit potential.
- Net payback period reduced from 4.02 months (CSS) to 3.76 months (MSS).
- Innovative fin design with waste materials provides a sustainable, low-cost alternative to conventional nanomaterials and metals.

### Limitations and future scope

The current work was conducted under clear-sky conditions and evaluated a small-scale unit (0.25 m^2^ basin area). Long-term performance under variable weather conditions, saline feedwater quality, and scaling/fouling effects were not explored.

Future research should investigate:

- Larger-scale prototypes with integrated thermal storage to extend night-time productivity.
- Hybridization with photovoltaic or waste-heat recovery systems for energy self-sufficiency.
- CFD analysis of a single-sloped solar still with the integration of hyperbolic fins and TWP nanocoating.

## Data Availability

The data used in the current study are within the manuscript itself.

## Abbreviations

TWP: Toner waste powder
SSWCHF: Solar still with coated hyperbolic fins
CSS: Conventional solar still
MSS: Modified solar still
FE-SEM: Field emission scanning electron micrographs
XRD: X-ray diffraction
PAC: Primary annual charge
CCP: Carbon credit production
NPBT: Net pay back time

## List of symbols

*F*_*e, bw−ig*_: Fractional exergy for evaporation.
*F*_*c, bw−ig*_: Fractional exergy for convection
$\:{Ex}_{e,bw-ig}$: Evaporation exergy value for water and glass cover (W/m^2^ K)
$\:{Ex}_{c,bw-ig}$: Convection exergy value for water and glass cover (W/m^2^ K)
$\:{Ex}_{ti}$: Total heat transfer
$\:{h}_{e,bw-ig}$: evaporative heat-transfer coefficient (W/m^2^ K)
$\:{h}_{c,bw-ig}$: convective heat transfer coefficient (W/m^2^ · K)
$\:{h}_{t}$: is the total heat transfer coefficient
$\:{A}_{bw}$: basin area of water (m^2^)
$\:{A}_{g}$: inner glass cover surface (m^2^)
$\:{T}_{bw}$: Basin water temperature (°C)
$\:{T}_{ig}$: Basin water temperature (°C)
$$\:{T}_{a}$$: Ambient temperature (°C)
$\:Ts$: Temperature of sun
$$\:{\eta\:}_{D}$$: Energy Efficiency
$\:{m}_{D}$: Daily freshwater mass (kg)
$\:L$: Latent heat of vaporization
$\:{I}_{t}$: Total solar radiation (W/m^2^)
$$\:{\eta\:}_{exe}$$: Exergy efficiency
$\:{Ex}_{out}$: exergy output value
$\:{Ex}_{in}$: Exergy input value
$\:\varphi\:{ex,co}_{2}$: Exergo - Exergo-environmental value
$\:Zc{o}_{2}$: Average carbon price
*R*_*ex*_: Value of economic exergy
*l *: Life of solar still
